# Deciphering the Therapeutic Mechanisms of Wuzi Ershen Decoction in Treating Oligoasthenozoospermia through the Network Pharmacology Approach

**DOI:** 10.1155/2021/5591844

**Published:** 2021-08-06

**Authors:** Mingrui Hu, Yuanyuan Zhong, Wei Xiao, Yang Wang, Tao Tang, Shunshun Wang, Hanjin Cui, Teng Li, Jiekun Luo

**Affiliations:** ^1^Institute of Integrative Medicine, Department of Integrated Traditional Chinese and Western Medicine, Xiangya Hospital, Central South University, Changsha 410008, China; ^2^Postpartum Health Care Department, Hunan Provincial Maternal and Child Health Care Hospital, Changsha 410008, China

## Abstract

**Background:**

Infertility affects approximately 15% of couples around the world, and male factors are accounted for 40–50%. Oligoasthenozoospermia is the most common reason for male infertility. Unfortunately, effective drug therapy is still lacking except for assisted reproductive technology (ART). Previous researchers found that Wuzi Ershen decoction (WZESD) can increase sperm count, enhance sperm vitality, and improve semen quality. However, the pharmacological mechanisms remain unclear.

**Methods:**

In this study, we screened compounds and predicted the targets of WZESD based on the TCMSP and BATMAN-TCM database combined with literature searching in the PubMed database. We obtained proteins related to oligoasthenozoospermia through GeneCards and submitted them to STRING to obtain the protein-protein interaction (PPI) network. Potential targets of WZESD were mapped to the network, and the hub targets were screened by topology. We used online platform Metascape and Enrichr for GO and KEGG enrichment analyses. AutoDock Vina was utilized for further verification of the binding mode between compounds and targets.

**Results:**

Totally, 276 bioactive compounds were obtained and targeted 681 proteins. 446 oligoasthenozoospermia disease-specific proteins were acquired, and further bioinformatics analysis found that they were mainly involved in the formation of gametes, meiosis, and sperm differentiation. Protein interaction network analysis revealed that target proteins of WZESD were associated with oligoasthenozoospermia disease-specific proteins. The 79 targets of disease-specific proteins, which were anchored by WZESD, mainly participate in the cellular response to the organic cyclic compound, regulation of the apoptotic process, nitricoxide biosynthetic and metabolic process, oxidative stress, and protein phosphorylation regulation, which are the causes for oligoasthenozoospermia. Molecular docking simulation further validated that bioactive compounds originated from WZESD with targeted proteins showed high binding efficiency.

**Conclusions:**

This study uncovers the therapeutic mechanisms of WZESD for oligoasthenozoospermia treatment from the perspective of network pharmacology and may provide a valuable reference for further experimental research studies and clinical applications.

## 1. Introduction

Infertility, defined as the inability to achieve a clinical pregnancy within 12 months of regular unprotected intercourse, is estimated to affect 15% of all couples globally [1]. Male factor is believed to account for 50% of infertile couples. It has been exponentially increasing in recent years due to a comprehensive evaluation of reproductive male function and improvements in diagnostic tools [2]. More than 40% of infertile men are diagnosed with oligoasthenozoospermia, and their poor sperm quality is considered as one of the major causes of infertility [3]. Genetic abnormity (Klinefelter syndrome, microdeletion on the Y chromosome), endocrine disorders (primary or secondary hypogonadism), testicular dysfunction (heat, drug, or radiation therapy), and infections (mycoplasma and ureaplasma) are the main causes for poor sperm quality [4–6]. However, the etiology is still unknown in about 50% of cases [4]. Work and progress on medical treatment of this syndrome have been neglected and ignored for the development of assisted reproductive technology (ART) [7]. Eﬀective pharmaceutical therapy for oligoasthenozoospermia is lacking [4]. Andrologists tend to search for potential novel drugs from the traditional Chinese medicine (TCM) library to cure oligoasthenozoospermia.

TCM is a comprehensive medicinal system that has been used in clinical practice for thousands of years and plays an important role in health maintenance for people all over the world [8, 9]. Numerous studies have reported that Chinese herbs can significantly improve the quality and quantity of the sperm, such as *Cordyceps militaris* [10], Shao-Fu-Zhu-Yu-Tang [11], Wuzi Yanzong pill [12], and Wuzi Ersen decoction (WZESD) [13]. WZESD is originated from the Wuzi Yanzong (WZYZ) pill, which is one of the most commonly prescribed Chinese herbal formulas to treat male infertility [12]. This herb formula consists of 12 herbs: *Cuscutae Semen* (*CS, Tusizi*), *Lycii Fructus* (*LF, Gouqizi*), *Rubi Fructus* (*RF, Fupenzi*), *Schisandra chinensis* (*SC, Wuweizi*), *Plantaginis Semen* (*PS, Cheqianzi*), *Radix Salviae* (*RS, Danshen*), *Figwort Root* (*FR, Xuanshen*), *Rehmanniae Radix Praeparata* (*RRP, Shudihuang*), *Dried Radix Rehmanniae (DRR, Shengdihuang*), *Epimrdii Herba* (*EH, Yingyanghuo*), *Ophiopogon japonicus* (*OJ, Maidong*), and *Polygonati Rhizoma* (*PR, Huangjing*). Our previous research found that WZESD can increase sperm count, enhance sperm vitality, and significantly improve semen quality and showed better efficiencies than the WZYZ pill in clinical practice [13]. However, the complexity of herbal ingredients, unknown targets in the human body, and complex interactive biological systems make it more difficult for herbal medicine research studies and restrict the application of TCM in the world [14]. It is critical to develop a novel method to deeply clarify the synthesized pharmacological mechanisms of WZESD for oligoasthenozoospermia treatment.

Network pharmacology was first described in 2007 by Andrew L. Hopkins, a professor at Dundee University in the United Kingdom [15]. It is an approach to drug design that encompasses systems biology, network analysis, connectivity, redundancy, and pleiotropy and offers a new framework for thinking about how to innovate drug discovery [16]. At the same time, Professor Shao Li proposed the “TCM network pharmacology” concept and established a network-based TCM research strategy [17]. Coinciding with the holistic and systemic characteristics of TCM, network pharmacology is expected to bridge the gap between TCM and modern medicine [18] and has become a flourishing field in TCM modern research studies along with the rapid progress of bioinformatics [19]. Based on genomics, proteomics, and metabolomics, it can research the essence of TCM and the function of herbal ingredients in a holistic way [20]. Successful attempts on TCM study have been achieved by network pharmacology such as Qing-Luo-Yin [21], Ma-Huang Decoction [22], Xuefu Zhuyu Decoction [23], and *Platycodon grandiflorum* [24].

In this paper, an integrated approach including active ingredient screening, target prediction, network construction, and analysis with molecular docking was used to reveal potential drug targets related to oligoasthenozoospermia, active compounds from WZESD, and their pharmacological mechanisms of action for oligoasthenozoospermia therapy. The present work may provide a valuable reference for further experiment research studies and clinical applications of WZESD for oligoasthenozoospermia treatment.

## 2. Materials and Methods

### 2.1. Database Construction

Candidate bioactive ingredients of 12 herbs in WZESD were screened from the Traditional Chinese Medicine Systems Pharmacology database (TCMSP, http://lsp.nwu.edu.cn/tcmsp.php) [25], the BATMAN-TCM [26, 27] (http://bionet.ncpsb.org/batman-tcm/) database, and literature searching in the PubMed database. TCMSP database is a unique systems pharmacology platform designed for herbal medicines. The newly developed TCMSP provides up-to-date, quantitative, and system information about TCM ingredients, ADME-related properties, targets, and diseases [28]. The newest version of TCMSP comprises 510 effective herbal entries registered in the Chinese Pharmacopoeia with more than 31,000 ingredients, which spread over 18 different drug classes [29]. BATMAN-TCM database is the first online bioinformatics analysis tool specially designed for the research of molecular mechanisms of TCM based on TCM ingredients' target prediction and subsequent network pharmacology analyses [26]. The structures of these ingredients were saved as a mol2 format for further analysis. Chem3D Pro 14.0 was employed to optimize these molecules and minimize the energy.

Active ingredient screening including absorption, distribution, metabolism, and excretion (ADME) evaluations of drugs is crucial in drug development and discovery [30]. Because biological experiments are time-consuming and of high cost, the identification of ADME properties by computational methods has now become an inevitable choice in pharmaceutical research. Here, 2 ADME-related models, including the evaluation of oral bioavailability (OB) and drug-likeness (DL), were calculated to screen the potential bioactive ingredients of WZESD. OB represents the percentage of an orally administered dose of unchanged drug that reaches the systemic circulation and is one of the most important pharmacokinetic parameters [31]. Poor OB is usually the main reason for the failure of drug discovery, especially for TCM which in most cases are oral administration. DL index is a qualitative concept used in drug design for estimating the “druggability” of a substance calculated using the Tanimoto coefficient [32, 33]. The evaluation of DL can facilitate screening for excellent compounds and increase the hit rate for the candidate drugs. For screening in the TCMSP database, OB ≥ 30% (a suggested criterion by the TCMSP database) and DL ≥ 0.18 (mean DL value for all DrugBank compounds) were regarded as the threshold for screening possible candidate drugs presently. At the same time, we also screened the BATMAN-TCM database and pieces of literature in the PubMed database with the Latin name of each herb for the candidate compounds.

### 2.2. Target Prediction

Drug-target mappings were obtained from three sources. Experimental validated drug-target pairs were retrieved from the HIT database [34]. For those ingredients without validated targets, an in-house developed model SysDT was used which efficiently integrated the chemical, genomic, and pharmacological information for drug targeting and discovery with the random forest (RF) and support vector machine (SVM) algorithm [35]. This method displays the incredible performance of prediction for drug-target interactions, with a concordance of 82.83%, a sensitivity of 81.33%, and a specificity of 93.62%, respectively [28]. The information for the targets based on the above 2 methods was collected from the TCMSP database. A similarity-based method on the basis of the BATMAN-TCM database was employed to predict potential targets of TCM ingredients, the core idea of which was to rank potential drug-target interactions based on their similarity to the known drug-target interactions [26]. UniProt [36] (http://www.uniprot.org/) was applied to obtain the official name of the predicted targets.

### 2.3. Oligoasthenozoospermia-Specific Protein Collection

Information on oligoasthenozoospermia-associated target genes was collected from GeneCards: the human gene database [37] (http://www.genecards.org/, ver. 4.8.2), and only “*Homo sapiens*” proteins linked to oligoasthenozoospermia were selected. GeneCards is a searchable, integrative database that automatically integrates gene-centric data from ∼125 web sources, including genomic, transcriptomic, proteomic, clinical, and functional information. Furthermore, these target genes were submitted to STRING [38, 39] (https://string-db.org/) to generate the protein interacting network. The STRING database aims to collect, score, and integrate all publicly available sources of protein-protein interaction information and to complement these with computational predictions and then achieve a comprehensive and objective global network, including direct (physical) as well as indirect (functional) interactions [40].

### 2.4. Molecular Docking

AutoDock Vina [41] was used in this study to evaluate the potential binding mode between bioactive compounds and putative targets; Discovery Studio 4.5 Client, BIOVIA [42], was applied to analyze the docked structures. The crystal structure of the target proteins of WZESD for oligoasthenozoospermia therapy was downloaded from the RCSB Protein Data Bank [43] (http://www.rcsb.org). The 3D chemical structures of bioactive ingredients were downloaded from the PubChem Compound database [44] or TCMSP database and submitted to minimize the energy by using the molecular mechanics-2 (MM2) force field in Chem3D Pro. The protein-ligand docking active site was defined by the location of the original ligand. Dimensions of the grids were set at 30 × 30 × 30 Ǻ in the *x*-, *y*-, and *z*-directions, with a spacing of 0.375 Ǻ between the grid points and the center placed at the active site of the original ligand crystallographic structures. All other docking and consequent scoring parameters used were kept at their default settings. The compound was regarded to be an efficient drug if the binding affinity was higher than that of the original ligand.

### 2.5. Network Construction and Analysis

To characterize the “multicomponent”, “multitarget” therapeutic mechanisms of herbal medicine for the treatment of oligoasthenozoospermia from a network target perspective, network construction was performed as follows:

1. Candidate compounds and candidate targets of WZESD were used to construct a candidate compound-candidate target (cC-cT) network
2. The PPI data obtained above were used to establish the oligoasthenozoospermia-specific protein interaction network
3. Potential compounds and putative targets from WZESD for oligoasthenozoospermia therapy were used to build a potential compound-potential target (pC-pT) network
4. Compounds and targets through molecular docking validation were used to construct a compound-target (C-T) network

All networks were generated and analyzed by an open source of bioinformatics package for biological network analysis and visualization, Cytoscape 3.5.1 [45]. Two topological parameters, degree and betweenness centrality, were calculated for the obtained networks which imply the significance of a node. The top 10 compounds and targets based on the 2 parameters were considered as key components and core proteins.

### 2.6. Bioinformatics Analysis

Gene Ontology (GO) analysis is a major bioinformatics tool for annotating genes and gene products, and it aims to unify the representation of gene and gene product attributes across all species [46]. The Kyoto Encyclopedia of Genes and Genomes (KEGG) [47] is a collection of databases containing advanced functional information for the systematic analysis of gene functions, biological pathways, diseases, drugs, and chemical substances. For GO and KEGG pathway analyses, the Metascape online tool [48] (http://metascape.org) and “Enrichr” platform [49] (http://amp.pharm.mssm.edu/Enrichr/) were used to identify the predominant biological processes (BP) and KEGG pathways regulated by WZESD.

## 3. Results

Thousands of years' clinical practices in herbal medicine have proven the in vivo efficacy and safety of herbal medicines [50], despite their mechanisms of action being generally unknown [51]. Fortunately, advances in systems biology and medicine have allowed the application of the systems pharmacology approach in the study of the herbal medicine. Unlike conventional medicine in which drugs are studied and used isolated, herbal medicine typically integrates several medicinal herbs which contain multiple chemical compounds that show more efficient therapeutic effects than an isolated single constituent [52]. In our work, a novel systems pharmacology approach integrated with polypharmacology and network biology was applied to uncover the therapeutic mechanisms of WZESD from a systematic level.

### 3.1. Bioactive Compound Screening and Target Prediction for WZESD

**Figure 1:**
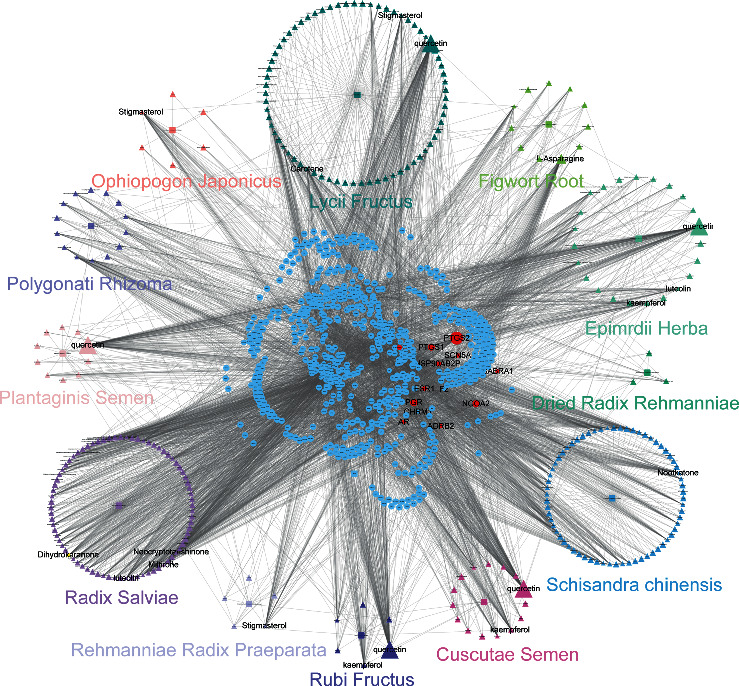
Candidate compound-candidate target (cC-cT) network of WZESD. Squares: herbs; triangles: candidate compounds for herbs of WZESD; circles: candidate targets predicted. The size of the node is proportional to the value of the degree, respectively.

A total of 276 candidate compounds were identified in WZESD, including 66 in SC, 10 in PS, 69 in LF, 15 in CS, 6 in RF, 77 in RS, 11 in FR, 3 in DRR, 5 in RRP, 14 in PR, 6 in OJ, and 23 in EH, respectively (Table S1). The 276 candidate compounds yield 681 candidate targets (Table S2), and the connections between them reach up to 5055. The candidate compounds and candidate targets were submitted to generate the cC-cT network (Figure 1), and details are depicted in the following part.

### 3.2. Candidate Compound-Candidate Target (cC-cT) Network Construction and Analysis

As shown in Figure 1, the network consists of 998 nodes and 5055 edges, including 12 herbs (squares), 276 compounds (triangles), and 681 targets (squares). The numbers of candidate targets in SC, PS, LF, RF, CS, RS, FR, DRR, RRP, PR, OJ, and EH were 204, 162, 388, 215, 178, 238, 178, 20, 20, 134, 41, and 216, respectively (Table S3). Although the number of targets in each herb was different, they overlapped dramatically in the 12 herbs indicating that different ingredients in WZESD shared common or similar targets with synergistic effects. The duplicated target proteins were removed, and 681 unique terms were retained. Much of the compounds possessed more than one target, and a target was anchored by more than one compound, revealing the “multicompound”, “multitarget” therapeutic mechanism of WZESD. Two centrality indicators, degree and betweenness, were calculated in this network. Different centralities reflect different importance of nodes in a network from different angles. The top 10 compounds and targets through network analysis are shown in Tables 1 and 2. Quercetin, L-asparagine, kaempferol, and stigmasterol were predicted as the major active compounds of WZESD. The proteins including PTGS2, NCOA2, PGR, PTGS1, and HSP90 were predicted as essential pharmacological proteins for the therapeutic effects of WZESD.

### 3.3. Analyses on the Oligoasthenozoospermia-Based Specific Protein Interaction Network

**Figure 2:**
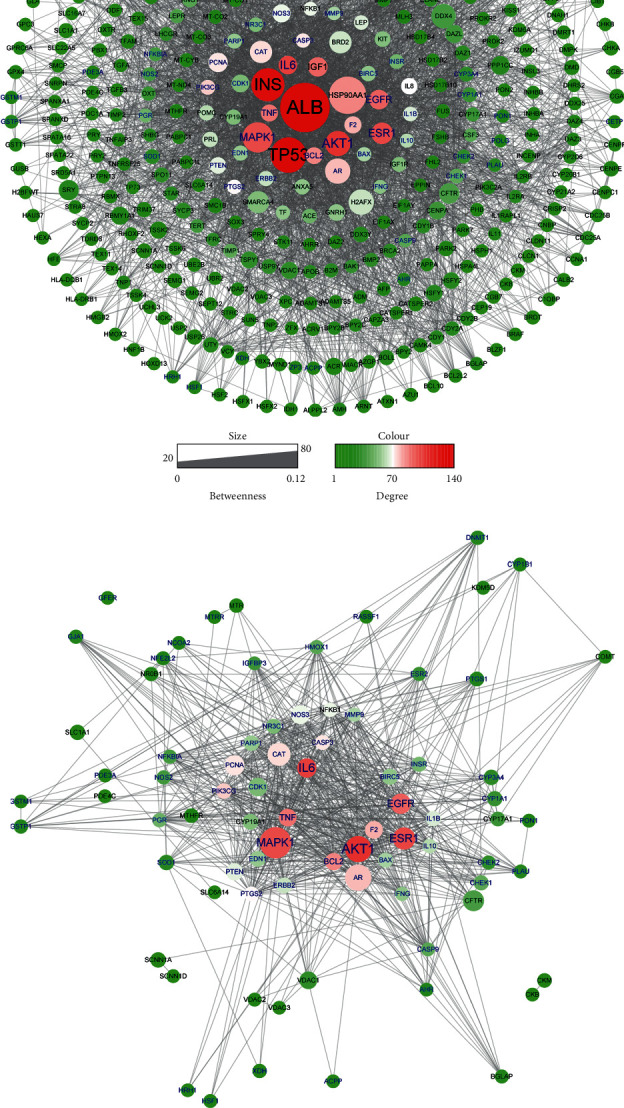
Oligoasthenozoospermia-related protein interaction network. (a) Total protein-protein interaction (PPI) network for oligoasthenozoospermia. (b) 79 candidate protein targets of WZESD screened for oligoasthenozoospermia therapy. The size and color of the node are proportional to the value of betweenness and degree, respectively.

446 target genes related to oligoasthenozoospermia were searched in the GeneCards database (Table S4) and further submitted to the STRING database (34 proteins were not recognized) to construct the protein-protein interaction (PPI) network (Figure 2(a)). This network contains 412 nodes and 4297 edges. The size and color of the node are proportional to the value of betweenness and degree (Table S5), respectively. The core proteins through network analysis were ALB, TP53, INS, AKT1, and MAPK1 (Table 3) which indicated the essential role of these proteins in the pathophysiology process of oligoasthenozoospermia. Seventy-nine proteins in this network were anchored by WZESD and further discussed in the following part (Figure 2(b)).

### 3.4. Co-Bioinformatics Analysis for Targets of WZESD and Oligoasthenozoospermia-Specific Proteins

**Figure 3:**
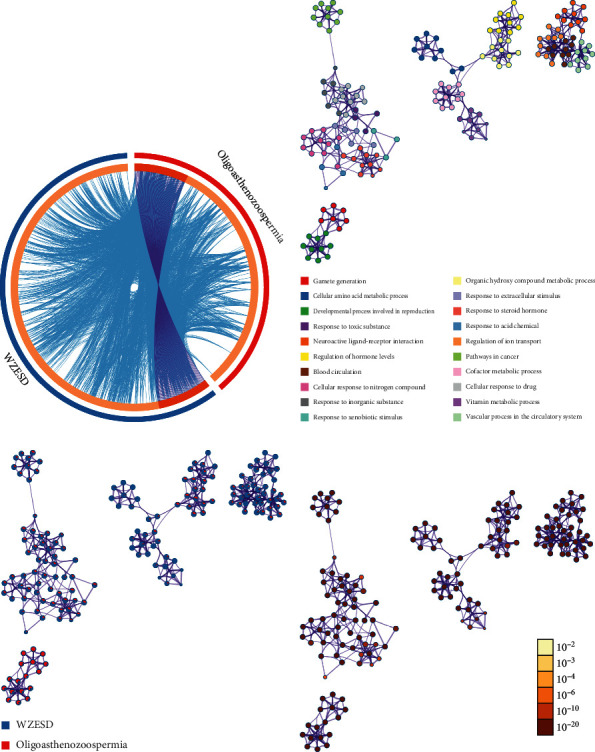
Co-bioinformatics analysis for targets of WZESD and oligoasthenozoospermia-specific proteins. (a) Circos plot for the 2 groups of proteins. Purple lines link the same proteins that are shared by multiple lists. Blue lines link different proteins where they fall into the same ontology term. (b) Enrichment network analysis for the union of the 2 groups of proteins. Each term is represented by a circle node, where its size is proportional to the number of input proteins that fall into that term, and its color represents its cluster identity. Terms with a similarity score >0.3 are linked by an edge (the thickness of the edge represents the similarity score). (c) The same enrichment network has its nodes displayed as pies. Each pie sector is proportional to the number of hits originated from the 2 lists. (d) The same enrichment network has its nodes coloured by the *p* value, as shown in the legend. The darker the color, the more statistically significant the node.

To explore the pharmacology mechanism of WZESD for oligoasthenozoospermia treatment, the relationship between WZESD targets and oligoasthenozoospermia-specific proteins was analyzed (Figure 3). 79 proteins were overlapped in the 2 groups of protein lists (Figure 3(a)). All the selected targets of WZESD and oligoasthenospermia-specific proteins were collected for enrichment network analysis, and most of the targets of WZESD could fall into GO items with the same statistical significance as oligoasthenozoospermia-specific proteins (Figures 3(b)–3(d)), indicating the strong function association between the two comparison cohorts. Gamete generation (GO: 0007276), the developmental process involved in reproduction (GO: 0003006), regulation of hormone levels (GO: 0010817), etc., were the top 10 enriched GO clusters.

### 3.5. Potential Compound-Potential Target (pC-pT) Network Construction and GO and KEGG Analyses

**Figure 4:**
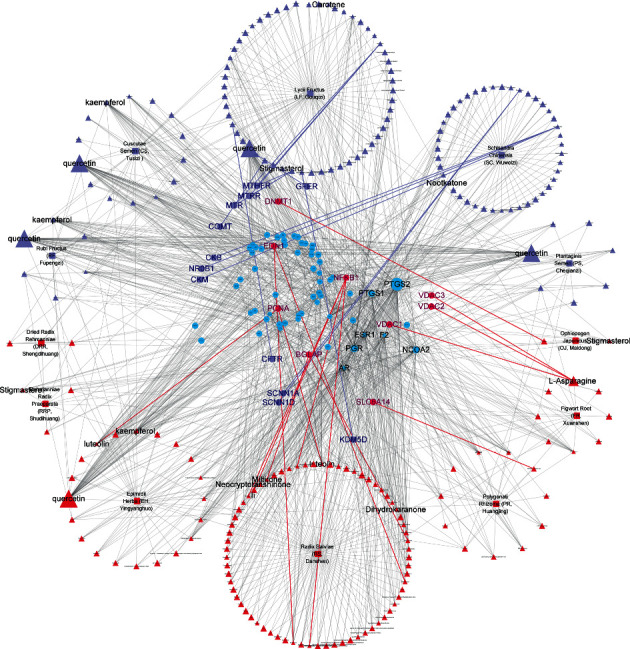
Potential compound-potential target (pC-pT) network of WZESD for treating oligoasthenozoospermia. The squares, triangles, and circles represent the herbs, potential compounds, and potential targets, respectively. LightSlateBlue: potential compounds and targets originated from the WZYZ pill. Red: potential compounds and targets derived from other herbs for WZESD. DeepSkyBlue: common compounds and targets in 2 comparison cohorts. The size of the node is proportional to the value of the degree.

To further investigate the therapeutic mechanism of WZESD for oligoasthenozoospermia treatment, the 79 overlapped target proteins were clarified in detail, and a pC-pT network was constructed (Figure 4). This network consists of 330 nodes (12 herbs, 213 potential compounds, and 79 potential targets) and 1390 edges. Compounds were manually divided into 2 groups of lists: compounds originated from WZYZ pill (LightSlateBlue) and compounds from other herbs of WZESD (red). The Wuzi Yanzong (WZYZ) pill is one of the most commonly prescribed Chinese herbal formulas for male infertility treatment. It was first recorded in the book called “*Xuan Jie Lu*” in AD 733 and spread around Northeast Asia over the next thousand years. *Cuscutae Semen* (*CS, Tusizi*), *Lycii Fructus* (*LF, Gouqizi*), *Rubi Fructus* (*RF, Fupenzi*), *Schisandra Chinensis* (*SC, Wuweizi*), and *Plantaginis Semen* (*PS, Cheqianzi*) are the main components of WZYZ pill. WZESD is originated from WZYZ pill. Based on WZYZ pill, *Radix Salviae* (*RS, Danshen*), *Figwort Root* (*FR, Xuanshen*), *Rehmanniae Radix Praeparata* (*RRP, Shudihuang*), *Dried Radix Rehmanniae* (*DRR, Shengdihuang*), *Epimrdii Herba* (*EH, Yinyanghuo*), *Ophiopogon japonicus* (*OJ, Maidong*), and *Polygonati Rhizoma* (*PR, Huangjing*) were added to enhance the efficacy. So, we compared the compounds and targets between the two groups. Seven compounds, including quercetin, campesterol, kaempferol, beta-sitosterol, sitosterol, gamma-sitosterol, and stigmasterol, were overlapped between the 2 groups (DeepSkyBlue triangles). 108 specific compounds (LightSlateBlue triangles) and 7 overlapped compounds (DeepSkyBlue triangles) from WZYZ pill anchored 70 potential targets (including 12 specific). However, 98 components (7 overlapped included) in the additional group targeted 67 potential proteins (including 9 specific proteins: EDN1, PCNA, DNMT1, SLC6A14, VDAC1, VDAC3, VDAC2, BGLAP, and NFKB1). Quercetin, kaempferol, luteolin, beta-sitosterol, and stigmasterol were the crucial potential active compounds, while PTGS1/2, PGR, NCOA2, ESR1, and AR were the core targets of WZESD for oligoasthenozoospermia therapy through network analysis (Tables 4 and 5).

For example, quercetin is a flavonoid that has been reported to possess strong antioxidant properties and is an effective free radical scavenger, and it showed positive effects on multiple functional parameters of spermatozoa, including viability and motility [53]. Kaempferol is another kind of flavonoid, and both quercetin and kaempferol can reduce the DNA damaging in the human sperm caused by reactive oxygen species (ROS) [54]. Luteolin can protect mice from severe acute pancreatitis through anti-inflammatory and antioxidant effects [55]. For target analysis, prostaglandin G/H synthase 1/2 (PTGS1/2), namely, cyclooxygenase-1/2 (COX-1/2), is the key enzyme in the conversion of polyunsaturated fatty acids and arachidonic acid to prostaglandin (PG). COX-2 has been demonstrated to be upregulated in infertility men [56], and treatment with the COX-2 inhibitor can improve sperm motility and morphology as well as increase the pregnancy rate in infertile males [57]. Estrogen receptor 1 (ESR1) is one of the main nuclear hormone receptors for estrogen and plays an important role in the regulation of spermatogenesis [58]. Efferent ductules and epididymal functions are dependent on estrogen signaling through ESR1, whose loss impaired ion transport and water reabsorption, resulting in an abnormal sperm [59]. Androgen receptor (AR) is essential for normal male reproductive development and function. AR signaling is required for the maintenance of spermatogonial numbers, blood-testis barrier integrity, completion of meiosis, adhesion of spermatids, and spermiation [60].

**Figure 5:**
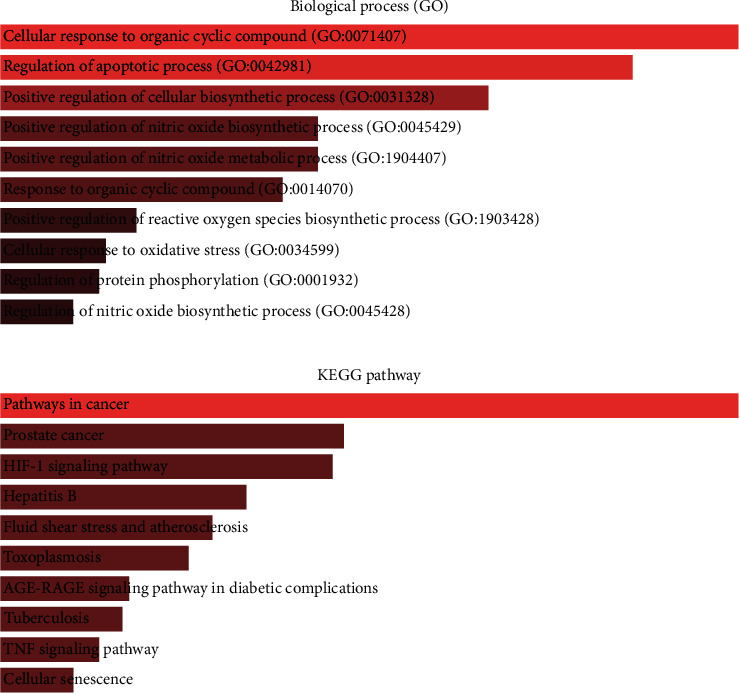
GO and KEGG pathway analyses for the 79 target proteins via the online “Enrichr” platform (http://amp.pharm.mssm.edu/Enrichr/).

GO and KEGG analyses were conducted via the online “Enrichr” platform to further explain the pharmacological mechanisms [61] (Figure 5). The 79 potential targets mainly participate in nitric oxide biosynthetic and metabolic process, apoptosis, and regulation of reactive oxygen biosynthetic process, which were strongly associated with oligoasthenozoospermia (Figure 5(a)). Reactive oxygen species (ROS) are an integral component of sperm developmental physiology, capacitation, and function. Elevated ROS levels, from processes such as infection or inflammation, can be associated with aberrations of sperm development, function, and fertilizing capacity [62]. The oxidative damage targets all cell components, reducing sperm motility and mitochondrial activity [63]. Therapy with ROS scavengers can significantly improve the in vitro function of human spermatozoa and may be beneficial in infertility patients [64, 65]. Apoptosis plays an important role in regulating spermatogenesis of various mammalian species, including humans [66]. This process of regulated cell death serves several important functions in the testis, a few of which include maintaining appropriate germ cells to Sertoli cell ratio, removing defective germ cells, and maintenance of overall quality control in sperm production [67]. However, high rates of apoptosis have been reported in testicular biopsies from infertile men [68]. Inflammatory disease has been established to affect male reproductive function and fertility. Male accessory gland infections account for almost 15% of all cases of male infertility seen in infertility clinics [69].

The top 10 enriched KEGG pathways were ranked in the descending order using a combined score as follows: pathways in cancer, prostate cancer, HIF-1 signaling pathway, etc. (Figure 5(b)). We found that pathways in cancer was the most significant enrichment term for the 79 target proteins; others such as hepatitis B, toxoplasmosis, and apoptosis share several same target genes with cancer-related pathways. This implies the crosstalk among different pathways in the complex molecular biological network. Many pathways such as the Wnt signaling pathway, PI3K-AKT signaling pathway, and MAPK signaling pathway are the main part of the cancer signaling pathway, which results in sustained angiogenesis, apoptosis evading, and cell proliferation. To a certain degree, there are several common pathways between the two different pathophysiological processes of spermatogenesis and tumorigenesis. The regulation of these signaling pathways by WZESD may be the therapeutic mechanism for oligoasthenozoospermia treatment.

### 3.6. Molecular Docking Validation

**Figure 6:**
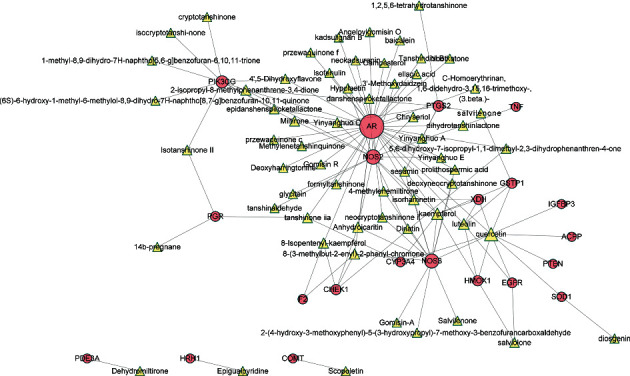
C-T network through molecular docking validation. 61 potential compounds (yellow triangles) are interacting with 21 potential targets (red circles) of WZESD. The size of the node is proportional to the value of the degree.

**Figure 7:**
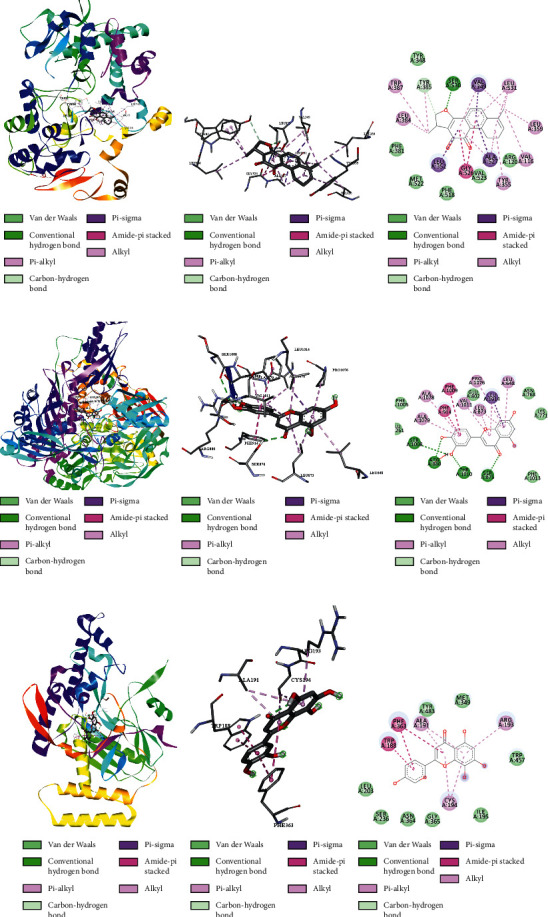
Predicted binding mode within the active site of the drug-target complexes obtained from AutoDock Vina. (a–c) PTGS2-1,2,5,6-tetrahydrotanshinone. (d–f) XDH-luteolin. (g–i) NOS2-hypolaetin (the PDB ID of PTGS2, XDH, and luteolin is 3NT1, 1N5X, and 3E65. (a, d, g) Global view. (b, e, h) 3D partial view. (c, f, i) 2D view for the interaction). The proteins are presented as cartoon modes, and molecules are presented as ball and stick models. Active site amino acid residues are represented as lines.

To evaluate the reliability of the above drug-target interactions and to further predict the accurate binding modes, a molecular docking method with AutoDock Vina [41] was employed. Molecular docking is a computational method that attempts to predict the noncovalent binding of macromolecules or, more frequently, of a macromolecule (receptor) and a small molecule (ligand) efficiently. The crystal structures of the potential targets were downloaded from the RCSB Protein Data Bank (http://www.rcsb.org). The original ligand was split and redocked to the proteins, the same as the potential compounds. A compound was considered to be an efficient drug only when the binding affinity was higher than that of the original ligand. 39 potential targets and 180 potential compounds were employed for molecular docking (Table S6). Finally, 61 potential compounds showed better binding affinity with 21 potential targets and formed 120 pairs of compound-target interactions (Figure 6). AR, NOS2/3, PIK3CG, and PTGS2 were the top 5 targets bound by active compounds through molecular docking validation. The detailed docking result is shown in Table S7. Three proteins, including PTGS2, XDH, and NOS2 with corresponding compounds that have the highest binding affinity, were used to display the exact binding mode (Figure 7). 1,2,5,6-Tetrahydrotanshinone was located in the binding pocket of PTGS2 and formed one conventional hydrogen bond by interacting with the key amino acid SER-530. Additionally, van der Waals with TYR-348, PHE-381, and ARG-120, pi-sigma with VAL-349, LEU-352, and ALA-527, and amide-pi stacked with GLY-526 were found in the active site which helped in the stabilization of the compound at the binding site (Figure 7(a), the global view; Figure 7(b), 3D partial view; Figure 7(c), 2D view for the interaction). In the active site of XDH, SER-876, ARG-880, SER-1008, and THR1010 formed 4 solid conventional hydrogen bonds with luteolin. Besides, other noncovalent bonds such as van der Waals, carbon-hydrogen bond, and amide-pi stacking were also formed in the binding site and helped in the stability of the complex (Figure 7(d), the global view; Figure 7(e), 3D partial view; Figure 7(f), 2D view for the interaction). However, there were no hydrogen bonds found in the interaction between NOS2 and hypolaetin, but van der Waals with ILE-195, LEU-203, SER-236, etc., and amide-pi stacked with TRP-188 and PHE-363 were recognized in the active site and mediated the pharmacologic action (Figure 7(g), the global view; Figure 7(h), 3D partial view; Figure 7(i), 2D view for the interaction). Based on these findings, we consider that noncovalent bonds such as hydrogen bond, van der Waals, carbon-hydrogen bond, and amide-pi stacking play key roles in the protein-ligand recognition and stability, which may help determine the pharmacology activities.

## 4. Discussion

Infertility pertains to approximately 15% of sexually active couples, and male factor is present in approximately 40% of infertility cases [70]. Many factors such as age, infection, environmental pollutant, and endocrine disorder are the main cause for male infertility. The most common cause is oligoasthenozoospermia. A variety of medications have been developed to improve the sperm quality and treat male infertility such as follicle-stimulating hormone (FSH), antiestrogens, L-carnitine, and antioxidants, but showed limited curative effect, and made it still a controversial issue [71]. Thus, men with infertility seek for one or more alternative therapies such as herbal medicines or acupuncture [72]. Wuzi Yanzong (WZYZ) pill, a kidney-reinforcing Chinese herbal formula, has been widely used to treat the syndrome of kidney deficiency, including impotence, spermatorrhea, premature ejaculation, and male sterility. Many research studies have reported that WZYZ pill can treat oligoasthenozoospermia by increasing sperm concentration, improving sperm motility, enhancing the activity of the acrosomal enzyme, and decreasing the sperm DNA fragmentation index [12, 73]. However, the efficacy of WZYZ pill in treating male infertility remains a matter of debate [12].

WZESD, a TCM formula development from the WZYZ pill according to TCM theory, showed a more efficient therapeutic effect for male infertility [13], but the underlying therapeutic mechanism is still unclear. Furthermore, due to the complex nature of TCM, it remains difficult to unveil such holistic medicine by the current reductionism research strategies [74]. In the current study, a network pharmacology-based method, including active ingredients' screening, multiple drug-target prediction and validation, and network construction and analysis with molecular docking, was used to clarify the pharmacological mechanisms of WZESD for treating oligoasthenozoospermia. 115 compounds (including 7 overlapped) of WZYZ pill anchored 70 potential targets (including 12 specific proteins). However, 98 components (including 7 overlapped) in the additional group targeted 67 potential proteins (including 9 specific proteins). The 79 potential targets mainly participate in nitric oxide biosynthetic and metabolic process, apoptosis, and regulation of reactive oxygen biosynthetic process, which were strongly associated with oligoasthenozoospermia. Previous limited studies reported that WZYZ Pill can improve spermatogenesis, possibly through modulating the expression of the secretory proteins of Sertoli cells [75], intervene in Tip60-mediated apoptosis [73], and alleviate testicular damage via its antioxidation [76], which were consistent with our study results. Clinical trial studies have also confirmed that WZYZ Pill has obvious effects in improving the quality of male semen [77, 78] and elevating the semen volume and sperm density in infertility patients with low semen counts [79], but the active compounds, direct targets, and molecule mechanisms were not mentioned in their research studies. Our study can suggest more comprehensive mechanisms of therapeutic effects of WZESD in terms of compounds, target proteins, and pathways than manual reviews of the literature.

WZYZ pill is an ancient formula and has been widely used for the treatment of male infertility. However, changes with the environment, climate, and human through hundreds of years made it less efficient. “Syndrome differentiation” and “human-environment interrelation” are 2 of the most important concepts in TCM theory. So, we need to modify the herb composition to deal with the changes. WZESD, based on WZYZ pill, added 7 commonly used herb medicines, which played the role of tonifying the liver and kidney, nourishing Yin and promoting fluid, accelerating blood circulation, removing blood stasis and dredging collaterals, and oozing wet and drenching, better accommodated the changes, and showed enhanced therapeutic effects in clinical research. In the molecule level, more active compounds (91) and proteins (9) apart from WZYZ pill were found in WZESD. We also found that quercetin, kaempferol, luteolin, beta-sitosterol, and stigmasterol were the crucial potential active compounds, while PTGS1/2, PGR, NCOA2, ESR1, and AR were the core targets of WZESD for oligoasthenozoospermia therapy (Tables 4 and 5). In another network pharmacology study, 39 bioactive compounds and 40 targets of the WZYZ pill associated with spermatogenesis disorder were obtained, and protein-protein interaction identified TP53, TNF, AKT1, Bcl-XL, Bcl-2, and I*κ*BA as hub targets [79]. Compared with two studies, WZESD could provide more compounds and targets; it was more likely to play a role in the treatment of oligoasthenospermia through more pathways. The “multicompound”, “multitarget”, and “multipathway” therapeutic mechanisms were further validated and may explain the efficacy of WZYZ pill as well as why WZESD showed better effects than WZYZ pill at the molecule level.

The dominant paradigm in drug discovery is the concept of designing maximally selective ligands to act on individual drug targets. The increase in the rate of drugs failing in late-stage clinical development over the past decade has been concurrent with the dominance of the assumption that the goal of drug discovery is to design exquisitely selective ligands that act on a single disease target [16]. Many common diseases such as cancer and rheumatoid arthritis as well as male infertility are complex biological systems caused by multiple molecular abnormalities. Drugs that modulate a single target might not always yield the desired outcome even if they completely interdict the functions of their direct targets [80]. With the rapid progress of bioinformatics, systems biology, and polypharmacology, network-based drug discovery is considered a promising approach toward more cost-effective drug development [17]. Coinciding with the holistic and systemic characteristics of TCM, network pharmacology is expected to bridge the gap between TCM and modern medicine. Our study successfully deciphers the efficacy of WZESD for the treatment of oligoasthenozoospermia at a molecule level and from a holistic perspective via a network pharmacology approach, which may provide a valuable reference for further experiment research studies and clinical usage.

Two main limitations of this study should be pointed out: (1) experimental investigations of WZESD for oligoasthenozoospermia treatment were not conducted; (2) the ingredients of herbs screened by our method may also be partially missing out compounds that still have effects.

## 5. Conclusions

The therapeutic effects of herbal formulas in disease management have been demonstrated by clinical practice over thousands of years, and the efficacy of WZESD for treating oligoasthenozoospermia has been proved by clinical research studies. Our work uncovers the therapeutic mechanisms of WZESD for oligoasthenozoospermia treatment from the perspective of network pharmacology and may provide a valuable reference for further experiment research studies and clinical applications.

## Figures and Tables

**Table 1:** Top 10 candidate compounds of WZESD according to 2 centrality indicators.

Compounds	Degree	Compounds	Betweenness centrality
Quercetin	155	L-Asparagine	0.136243
L-Asparagine	97	Quercetin	0.048328
Neocryptotanshinone II	66	Epiguaipyridine	0.046678
Kaempferol	64	Clupanodonic acid	0.045057
Stigmasterol	59	Vitamin C	0.042922
Carotene	56	Riboflavine	0.039313
Miltirone	52	Carotene	0.038851
Luteolin	52	Stigmasterol	0.036878
Nootkatone	45	Neocryptotanshinone II	0.036227
Dihydrokaranone	44	Nootkatone	0.032663

**Table 2:** Top 10 candidate targets of WZESD according to 2 centrality indicators.

Protein	Degree	Protein	Betweenness centrality
PTGS2	117	PTGS2	0.08777186
PGR	76	PGR	0.04445812
NCOA2	75	PTGS1	0.0402169
PTGS1	73	RNASE1	0.03755925
ESR1	58	NCOA2	0.0311451
HSP90AB2P	58	AR	0.02706378
SCN5A	54	GABRA1	0.02468627
AR	53	ESR1	0.02400916
ADRB2	53	ADRB2	0.02027517
GABRA1	52	SCN5A	0.01794721

**Table 3:** Top 10 proteins of oligoasthenozoospermia-specific proteins according to 2 centrality indicators.

Proteins	Degree	Proteins	Betweenness centrality
ALB	143	ALB	0.11298229
TP53	135	HSP90AA1	0.07592846
INS	130	TP53	0.06982591
AKT1	110	INS	0.06260318
IL6	105	MAPK1	0.05384132
ESR1	102	AR	0.03846234
MAPK1	101	H2AFX	0.03797663
EGFR	95	AKT1	0.03794033
IGF1	94	DDX4	0.034775
TNF	91	CAT	0.0289976

**Table 4:** Top 10 potential compounds of WZESD for oligoasthenozoospermia therapy according to 2 centrality indicators.

Compounds	Degree	Compounds	Betweenness centrality
Quercetin	50	Quercetin	0.03918077
Kaempferol	27	Stigmasterol	0.0208998
Luteolin	24	L-Asparagine	0.01886399
Beta-sitosterol	16	Kaempferol	0.01436287
Baicalein	14	Beta-sitosterol	0.0143097
Isorhamnetin	13	Epiguaipyridine	0.01308015
8-(3-Methylbut-2-enyl)-2-phenyl-chromone	11	Luteolin	0.01237774
Anhydroicaritin	11	Nootkatone	0.01196325
Tanshinone IIA	11	Diosgenin	0.00997361
Paeonol	10	Vitamin B12	0.00906294

**Table 5:** Top 10 potential targets of WZESD for oligoasthenozoospermia therapy according to 2 centrality indicators.

Protein	Degree	Protein	Betweenness centrality
PTGS2	117	PTGS2	0.23041585
PGR	76	PGR	0.12652197
NCOA2	75	NCOA2	0.0913815
PTGS1	73	PTGS1	0.07917499
ESR1	58	ESR1	0.06231303
AR	53	F2	0.05831288
F2	51	AR	0.05691608
PIK3CG	35	CAT	0.04291953
CAT	23	PDE3A	0.01740412
PDE3A	23	PIK3CG	0.01281069

## Data Availability

All the data generated and analyzed during this study are included within this published article and its supplementary information files. The datasets supporting the conclusion in this study are available in a public database from TCMSP, BATMAN-TCM, PubChem Compound, GeneCards, UniProt, STRING, Metascape, and Enrichr.

## Supplementary Materials

Supplementary Materials Table S1: candidate compounds of each herb in WZESD. Table S2: detailed information for the 681 candidate targets of WZESD. Table S3: candidate compounds and their candidate targets for WZESD. Table S4: 446 target genes related to oligoasthenozoospermia obtained from GeneCards. Table S5: topological parameter for the generated oligoasthenozoospermia-specific protein interaction network. Table S6: the proteins and compounds performed for molecular docking. Table S7: molecular docking results of 61 potential compounds with 21 potential proteins.
